# Nucleic acid aptamer application in diagnosis and therapy of colorectal cancer based on cell-SELEX technology

**DOI:** 10.1038/s41698-017-0041-y

**Published:** 2017-11-14

**Authors:** Chan Chen, Shan Zhou, Yongqiang Cai, Faqing Tang

**Affiliations:** 1 Clinical Laboratory and Medical Research Center, Zhuhai Hospital of Jinan University, Zhuhai People’s Hospital, 519000 Zhuhai, Guangdong China; 2 Clinical Laboratory, Hunan Cancer Hospital & The Affiliated Cancer Hospital of Xiangya School of Medicine, Central South University, 410006 Changsha, China

## Abstract

Nucleic acid aptamers are a class of high-affinity nucleic acid ligands. They serve as “chemical antibodies” since their high affinity and specificity. Nucleic acid aptamers are generated from nucleic acid random-sequence using a systematic evolution of ligands by exponential enrichment (SELEX) technology. SELEX is a process of effectively selecting aptamers from different targets. A newly developed cell-based SELEX technique has been widely used in biomarker discovery, early diagnosis and targeted cancer therapy, particular at colorectal cancer (CRC). Combined with nanostructures, nano-aptamer-drug delivery system was constructed for drug delivery. Various nanostructures functionalized with aptamers are highly efficient and has been used in CRC therapeutic applications. In the present, we introduce a cell- SELEX technique, and summarize the potential application of aptamers as biomarkers in CRC diagnosis and therapy. And some characteristics of aptamer-targeted nanocarriers in CRC have been expatiated. The challenges and perspectives for cell-SELEX are also discussed.

## Introduction

Nucleic acid aptamers are a class of high-affinity nucleic acid ligands, which are selected through ssDNA or RNA binding a specific target molecule from a region library in vitro. In 1990, Tuerk and Gold found two high-affinity RNA ligands for T4 DNA ploymerase using interative selection rounds from randomized sequence pools and bound species amplification.^1^ This process of alternate cycles in vitro was termed systematic evolution of ligands by exponential enrichment (SELEX). Robertson and Joyce then used this techniques to screen and identify the first RNA enzyme.^2^ In the typical SELEX procedure, designing a random sequence library is complicated, it involves a 10^15^–10^16^ random sequence with the length of 20 to 40 bp. A SELEX experiment includes four steps: (1) screening the condition of incubation with target molecule; (2) selection of bound sequence; (3) elution of unbound species, and (4) amplification of the bound nucleic acids. However, aptamer possesses a high affinity and specific binding ability. An aptamer is superior to an antibody in clinical applications such as diagnosis, drug release and targeted therapy.

To date, using SELEX technology has successfully generated thousands of aptamers, which bind to specific targets including small molecules, metal ions, proteins, peptides, bacteria, virus, and live cells.^3–8^ Other, aptamers can bind to surface molecules and membrane proteins of live cells.^9^ Cancer cells possess various tumor-associated membrane proteins on their surface, various aptamers can target these protein moleculess.^10^ So, some aptamers targeting tumor-associated membrane proteins were generated for cancer detection and chemotherapy.^11^ Following cell-SELEX development, aptamers are widely applied in the molecular diagnosis and therapy against cancer.

Colorectal cancer (CRC) is the most frequently diagnosed cancer claiming approximately 700,000 lives every year.^12^ Presently, it is difficult to target distant metastases of CRC, and tumor metastasis is the main cause of CRC death.^13^ Since distant metastasis of CRC at stage IV, its five-year survival rate is much lower than that at stage I.^14^ Early diagnosis and targeted treatment are a main strategy to promote the five-year survival rate. Traditional biomarkers for CRC diagnosis, such as CEA and CA19-9 have poor specificity, and can’t be used to detect early stages. Aptamers as a specific targeting molecule are used to discriminate various receptors and biomarkers on CRC cell surface, and it may be used in CRC early diagnosis. In CRC therapy, targeted-drug delivery systems could help clinicians to reduce effects of chemotherapeutic drugs. Aptamer has affinity and selectivity, and nanostructure serves as a smart carrier for drug delivery. Combination of aptamer and nanotechnology has successful used been in CRC therapeutic and diagnostic applications. In current, a variety of nanomaterials have been used in CRC diagnosis and therapy, such as magnetic nanoparticles, polymers nanoparticles and silica nanoparticles.^15–17^ They have remarkable characteristics including chemical properties and controllable physical, high stability, and high carrier capacity.^18^ Herein, we focus on Aptamer application in CRC diagnosis and therapy, andaptamer-conjugated nanoparticles for CRC targeted drug delivery.

## Nucleic acid aptamers

Nucleic acid aptamers possess three-dimensional structures in which chemical reactions bind targets via van der Waals forces, hydrogen bonding, salt bridges, other electrostatic interactions, and shape complementarity.^19–22^ The dissociation constants (Kd) of nucleic acid aptamers range from a pico to a nanomolar level. A substantial limitation of aptamers is that the unmodified nucleic acid is sensitive to serum nucleases.^23^ However, aptamer can be modified in vivo to enhance its bioavailability and stability.^24,25^ An aptamer can be partially or completely substituted with one or more modifications, and conjugated with functional molecules, such as 2′-amino pyrimidines, 2′-fluoro pyrimidines, 2′-O-methyl ribose purines, or polyethylene glycol (PEG).^26–33^ Aptamers are non-toxic and lack immunogenicity in comparison with antibodies. According to their chemical properties, aptamers are also referred to as “chemical antibodies”, highlighting their functional similarity to protein antibodies. In the antibody therapy against cancer, the antibody molecule is large and difficulty to penetrate into the tumor tissue. Aptamer has a flexible structure and its size is ~25-fold smaller compared with that of monoclonal antibody.^34^ Therefore, aptamer is superior to antibody in tumor accumulation and penetration in vitro and in vivo. Compared with antibodies, aptamers possess little to, no immunogenicity, and low toxicity in normal cell in vivo, these are important features for in vivo tumor imaging. The following illustrates a comparison between aptamer and antibody in cancer treatment (Table 1).^34^

**Table 1:** Diagnosis and treatment of aptamer versus antibody

Application	Aptamer	Antibody
Internalizing into target cell	Efficiently internalization	Poor internalize into tumors
Tumor sphere penetration	At least a 4-time better tumor penetration	Hardly penetration
Tumor imaging in vivo	A much better clinically signal -to-background ratio	More slower uptake in target sites
Drug delivery in vivo	4.3-Fold longer of sustainable signal	Lower retention in tumor sites

## Cell-SELEX technology

**Fig. 1:**
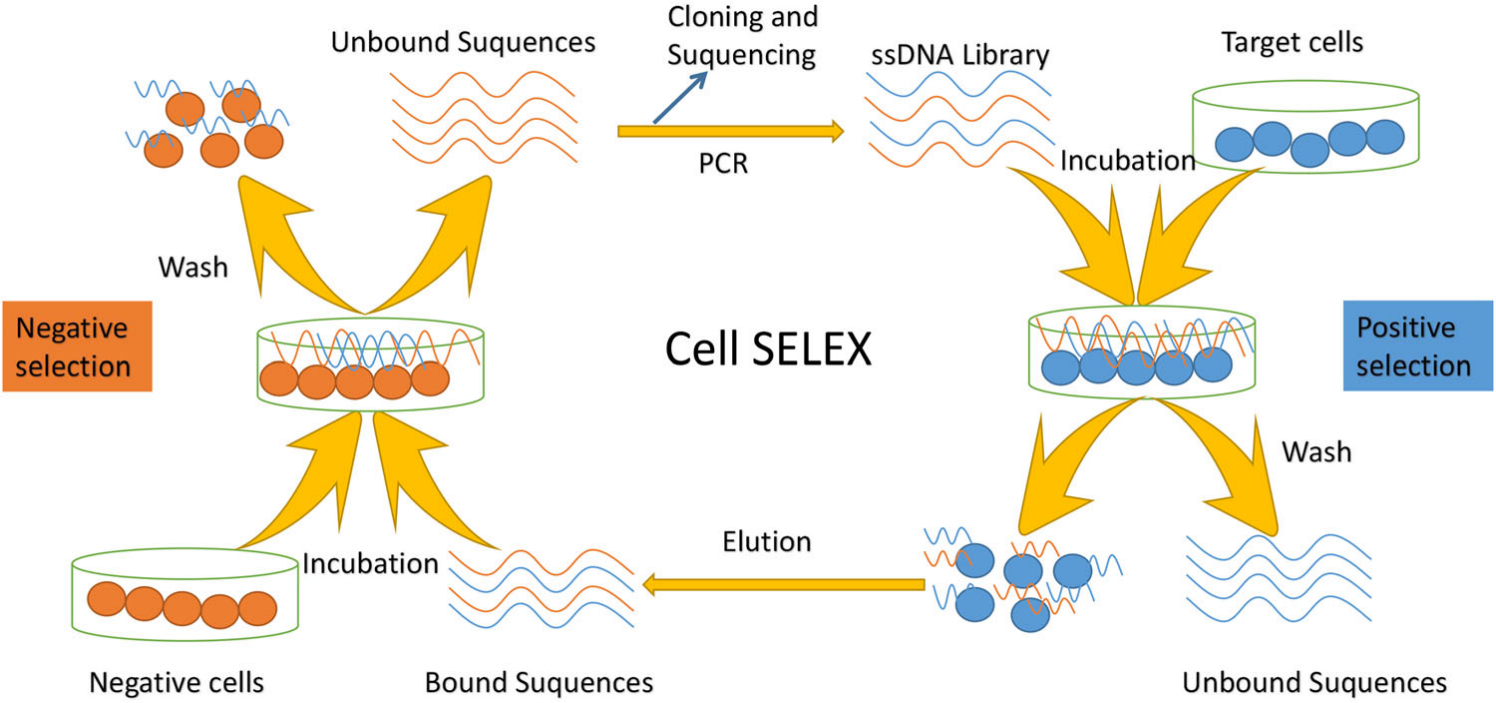
Schematic reprentation of specific aptamer selection using cell-SELEX. Cell-SELEX uses living cell as target. Aptamers bind with living cell membrane proteins. The procedure of cell-SELEX includes positive selection and negative selection. The positive selection is ssDNA library incubation with target cells, following the bound sequences are collected. The bound sequences are incubated with negative cell, and the unbound sequences were collected and served as the negative selection. The unbound nucleic acids are used for amplification, sequencing, and cloning. Aptamers are obtained after 10–15 alternate cycles

Cell-SELEX technology has been modified from the classic SELEX procedures. Cell-SELEX requires positive and negative selection in order to select an aptamer with greater affinity and specificity. Recent studies report, using specific cancer cell as targets and normal and noncancerous cell as negative-selection targets to screen aptamers, the aptamers specifically bind to the target cancer cells.^35^ Cell-SELEX is believed to be easy to handle, fast and reproducible. In cell-SELEX process, incubation, elution, and amplification are main steps for obtaining aptamers. Firstly, ssDNA pool is incubated with target cells. After incubation, the unbound ssDNAs are washed away, and the target cells bound with ssDNA were collected, and this bound DNAs are separated by heating the target cells. The bound DNAs are amplified using PCR, and the PCR product is incubated with streptavidin magnetic beads, and the bound DNAs in the PCR product were captured using streptavidin magnetic beads. This procedure is generally repeated 10–15 times, the enriched product is monitored by flow cytometry binding assays, finally, the specific aptamers are obtained^35^ (Fig. 1). In order to obtain the aptamers with high affinity and specificity, selection pressure should be gradually enhanced in incubation and elution strep, including decreasing ssDNA amount and reducing incubation time, as well as increasing wash times. In cell-SELEX, living cells are used as targets, cell culture and active receptor expressions are also critical for effective selection. In 1998, the first SELEX screening protocol was constructed for isolating high specificity aptamers, this study illustrated that aptamers have a great potential for combining with multiple targets and aid in dissecting complex biological systems.^36^ The cell subtractive SELEX technique was published in 2003,^37^ this technology was based on cell-SELEX and aimed to introduce a class of similar or homologous cells. As its development, the cell-SELEX technology exerts an increasing important role in screening for cancer biomarker.

In the traditional SELEX technology, proteins as targets had to be extracted and purified, however, the purified proteins had low solubility and yield, which limited their application. The cell-SELEX technology solves the above problems and has the following advantages: (1) Aptamers from whole living cells have an ability to characterize target cells and specifically recognize molecular signatures of different cell types. These aptamers are optimal molecular probes for specific recognition of target cells. (2) The living cell surface contains numerous different molecules, and these different molecules are served as targets for aptamers selection. The selected aptamers can selectively recognize corresponding targets. (3) Aptamers selected using cell-SELEX technology can recognize targets and be applied in biomarker discovery. These aptamers can bind to unknown molecules which are receptors and biomarkers in cell surface, some novel specific functions of aptamers may be discovered.^38^ Binding with cell surface receptors via signaling transduction pathway, aptamers can regulate cell signaling and aid in the discovery of new function.^39^ (4) Aptamer selected using cell-SELEX technology is a high-quality molecular probe for binding to target molecules in cell native environment and to identify targets for conjugation^40^ (Fig. 1). The optimal aptamer probes can specifically recognize target proteins, and found new potential markers for tumor diagnosis and therapy. Using B-cell Burkitt’s lymphoma cell line (Ramors cells), aptamer TD50 with strong affinity and excellent specificity was selected and identified. TD50 can recognize cell surface membrane proteins. After chemical modification, TD50 is an effective molecular probe, which will play an important role in the future medical diagnosis and treatment of lymphoma.^41^

Cell-SELEX technology can be used in molecular diagnosis, biomedical discovery, cell-specific therapeutics, and stem cell therapy. However, there remain several technical disadvantages and unsolved defects. The limitations of the cell-SELEX technology are as follows: (1) In aptamer screening process, cell death is occasionally encountered. Dead cells influence single-stranded DNA, resulting in failure of the selection process.^42^ (2) Since cell membrane surface is a diverse and structurally complex system, the process of aptamer selection is also complex and time consuming, which increases cost.^43^ (3) Separation and purification of membrane proteins remains challenging. Additionally, membrane proteins are oncealed, frequently resulting that target cells can’t be integrated.^44^ Currently, there is no valid approach to overcome these difficulties. Nevertheless, it is imperative to develop a new and effective method to improve screening efficiency and shorten screening time.

## Biomarker screening and identification using cell-specific aptamers

Identification of novel biomarkers offers a promise of early cancer detection, and is valuable in targeted clinical treatment. Several aptamers, binding to biomarkers on the surface of tumor cells, have been used in cancer diagnosis and therapy (Table 2). In 2003, DionA^40^ used the cell-SELEX technology to identify DNA aptamer GBI-10 that targets a glioblastoma-derived cell line U251. They used GBI-10 to screen tumor markers and obtained tenascin-C. Tenascin-C is widely used in tumor diagnosis and therapy.^45^ Using cell-SELEX selection, protein tyrosine kinase 7 (PTK7), a transmembrane receptor protein tyrosine kinase-like molecule, was identified in a T-cell acute lymphoblastic leukemia (T-ALL) cell line (CCRF-CEM).^46^ Specifically, aptamer sgc8c binds to PTK7 on the leukemia cell surface. This aptamer, conjugated with magnetic beads, was developed as a diagnostic tool and has been used in therapeutic approaches. Aptamer AS1411, HCHO7, and Apt-32 have been confirmed to specifically bind to the biomarkers on the surface of cancer cells. AS1411 was generated against nucleolin in the plasma membranes of cancer cells,^47^ its phase II clinical trials were achieved, and it has been used to treat patients with leukemia. AS1411 had been approved to selectively deliver the chemotherapeutic drug doxorubicin (DOX) into cancer cells.^47,48^ HCHO7 isolated using the cell-SELEX targets insulin-like growth factor 2(IGF II) on the surface of lung cancer cells, it has been effectively used in lung cancer therapy.^49^ Aptamer 32 was identified to bind to the epidermal growth factor receptor variant III (EGFRvIII) on the glioblastoma multiforme (GBM) U87Δ cell line. Additionally, aptamer 32 can also be endocytosed to deliver drugs or siRNA in GBM therapy.^50^

**Table 2:** Identification of aptamer targeting cancer cell-surface molecule using cell-SELEX technology

Target	SELEX method	Type DNA/RNA	Aptamer name	Application	Refs	Year
tenascin-C	Cell-SELEX	DNA	GBI-10	Tenascin-C-targeted aptamers using for diagnostic and therapeutic	40	2003
RET/MEN2A mutant	Cell-SELEX	RNA	D4	Aptamers inhibiting RET-dependent intercellular signaling pathway	51	2005
Nucleon	Cell-SELEX	DNA	AS1411	Dox delivery with aptamer-PEG conjugate	42,43	2007
PTK-7	Cell-SELEX	DNA	Sgc-8	Aptamers being effectively used as biomarker discovery	41	2008
IGF II	Cell-SELEX	DNA	HCH07	Aptamer probes being used for all lung cancer types of early detection studies	44	2008
HPV-16 E6/ E7	AC-SELEX	DNA	Apt-14	Aptamers used to identify new biomarkers related to HPV-associated cervical cancer	46	2012
HPV-16 E6/ E7	Cell-SELEX	RNA	C5	Aptamers internalizing into cells, being useful for delivering therapeutic agents to HPV-16 associated malignancies.	47	2013
CD133	Cell-SELEX	DNA	A4	Cancer stem cells targeting therapeutic resistance, tumor spread and angiogenesis.	52	2013
CD133	Cell-SELEX	RNA	Apt-CD133	Aptamers targeting cancer stem cells	53	2013
EGFRvIII	Cell-SELEX	DNA	Apt-32	Aptamers promising molecular probes for the diagnosis and treatment of glioblastoma multiform	45	2013
EpCAM	Cell-SELEX	DNA	EP166	Aptamers used as a stem cell marker or in both stem cell and cancer studies.	54	2014
STIP1	Cell-SELEX	DNA	TOV6	Aptamers as a new drug class to block important	55	2014
Ets1	Cell-SELEX	RNA	Apt-GNP	Aptamers used as a diagnostic marker for Ets1-overexpressing highly progressive tumors	56	2015
CD44/CD24	Cell-SELEX	DNA	MS03	Aptamers as promising molecular probe during diagnostic and therapeutic applications of breast cancer.	57	2015
ALLPL2	Cell-SELEX	Nuclease resistant RNA	SQ2	Pancreatic cancer diagnosis and therapy	58	2015
IGHM	Cell-SELEX	DNA	TD05	Aptamers conjugated with polymeric nanoparticles for cancer cell detection	35,48	2015
MRP1	HT-SELEX	RNA	Apa-MRP1	Binding MRP1-expressing tumors, and delivering CD28 costimulatory signal to tumor-infiltrating lymphocytes	59	2016
Cytokeratin 19	Cell-SELEX	DNA	LY-1	Aptamers as a promising molecular probe for metastatic HCC	60	2016
HER2	Cell-SELEX	DNA	HeA2_1 /HeA2_3	A new method for diagnosis and therapy of HER2 positive breast cancer	50	2016

Several DNA/RNA aptamers can specifically internalize into HPV-16 E6/E7 transformed human tonsillar epithelial cells.^51^ These aptamers are used to identify new biomarkers in carcinogenesis. Of them, RNA aptamers can be internalized for siRNA delivery to HPV-associated tumors, and are used in HPV-positive cancer therapy.^52^ Moreover, immunoglobulin U heavy chain (IGHM) is a membrane proteins expressed in Burkitt’s lymphomacell-surface.^40^ Aptamer TD50 was identified using the cell-SELEX target to IGHM. To increase the stability of aptamer-protein binding, aptamer TD50 was chemically modified with protoactive 5-dUI.^53^ The modified aptamer TD05 has been used to the diagnosis and therapy of Burkitt’s lymphoma. Human epidermal growth factor receptor 2 (HER2, also known as ErbB2), a tyrosine kinase receptor, is highly expressed in breast cancer.^54^ HeA2-1 and HeA2-3 were two aptamers targeting HER2 on the surfaces of human breast cancer cells (SKBR3).^55^ A novel diagnostic method for breast cancer was constructed based on specific carbon nanotube (CNT)-wrapped anti-HER2 aptamers. These two aptamers greatly improved the sensitivity and accuracy of breast cancer diagnosis.

## Aptamer-directed CRC biomarkers

In the clinic, an endoscopy is widely used for CRC diagnosis. The endoscopy inspection being positive is strong evidence in the CRC diagnosis. However, endoscopy can be traumatic, and many patients at the early stages will not accept this examination.^56^ Carcinoembryonic antigen (CEA), a type of tumor marker, is widely used in CRC diagnosis. But CEA expression lacks specificity, this limits its application in CRC detection.^57^ A new technology based on cell-SELEX can potentially benefit early diagnosis. Specific aptamers against CRC cell-surface molecules have been identified for biomarker discovery. SYL3C, a aptamer targeted-epithelial cell adhesion molecule (EpCAM), can potentially be applied as a molecular probe in CRC.^58,59^ EpCAM, an antigen first found in CRC, is used as a molecular biomarker for CRC.^60^ In 2014, Ying P confirmed that SYL3C specifically bound to CRC tissue sections, they used SYL3C to replace EpCAM-antibody in the molecular diagnosis of CRC.^61^ The metastatic stage of CRC remains generally incurable,^12^ highlighting the critical need to identify an aptamer. DHX9, also termed RNA helicase A or nuclear DNA helicase II, is overexpressed in CRC metastases. RNA aptamer S-1 can specially recognize DHX9 in CRC tumor cell lines 57X and 119X, aptamer S-1 can bind to DHX9 protein in CRC.^62^ Thus aptamer S-1 may serve as a diagnostic marker for CRC metastases.

## Aptamer-based targeted therapy for CRC

In CRC chemotherapy, many agents lack specificity to target tumor cell, and can damage normal cells. The aptamer targeting tumor cells is conjugated to nanoparticles to develop a nano-aptamer-drug delivery system. This drug delivery system dramatically increases the affinity and specificity of aptamer. Nano-aptamer-drug delivery system exhibits some significant advantages, such as simple and affordable manufacturing process without using organic solvents or potentially toxic ingredients, high stability, and high carrier capacity. Additionally, nano-aptamer-drug delivery system enables the controlled release of loaded drug, improves drug bioavailability.^18^ Aptamers as molecular probes should efficiently recognize cancer cell, and directly deliver chemotherapeutic drug to tumor cells. In following, we discuss applications of aptamer–nanomaterial complexes in CRC therapy.

### Aptamer–SPION conjugates for targeted drug delivery

**Fig. 2:**
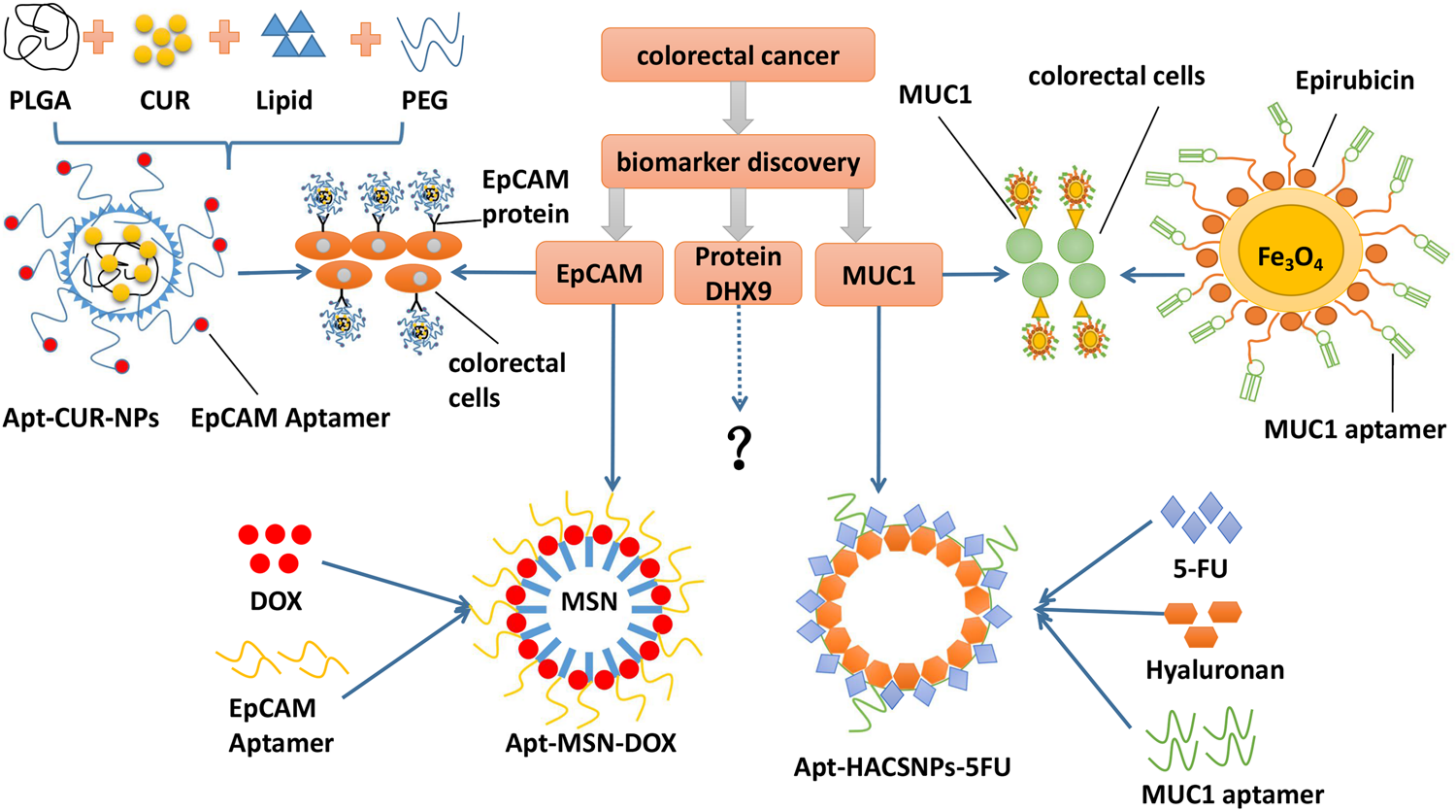
Schematic illustration of aptamer-based bioconjugate system for CRC targeted therapy. EpCAM, MUC-1, and DHX9 proteins serve as biomarkers against aptamer for CRC detection. Chemotherapy drugs loaded onto nanoparticle-aptamer bioconjugate are used for targeted drug delivery

MUC-1, a cell surface molecule, is expressed in various epithelial cancer, such as breast, colon, pancreatic and prostate.^63^ 5TR1 aptamer targets MUC1 and internalizes into colon tumors to specifically kill cancer cells.^64^ In cancer chemotherapy, the use of epirubicinis is limited by its toxicity, however, being conjugated with an aptamer, specific delivery of chemotherapeutic agents can reduce its toxicity effects (Fig. 2). In clinical diagnosis, super paramagnetic iron oxide (SPION) is a medical imaging tools, it has some advantages including low toxicity, low limit of detection and improved relaxation of proteins.^65^ SPION nanoparticle-aptamer bioconjugate with epirubicin (Epi-Apt-SPION) is used for magnetic resonance imaging (MRI), it can effectively detect tumor growth and therapeutic effect in vivo. Moreover, the Epi-Apt-SPION bioconjugate can reduce the viability of cancer cell.^66^ This indicates that targeted aptamers can serve as delivery vehicles to specifically transport chemotherapeutic drugs into colon cancer cells.

### Aptamer-PLGA nanoparticle conjugates for targeted drug delivery

To obtain a precise and efficacious drug delivery, active target nanotechnology-based drug delivery systems have been developed and play important roles in anticancer treatment.^67^ Curcumin has a potential anticancer function, however, it is limited by poor delivery and low systemic bioavailability in vivo.^68^ The nanoparticle drug delivery system greatly increases curcumin application in the clinical areas. Some efficacy trials show that curcumin can be delivered to colorectal cancer cells via aptamer-functionalized PLGA-lecithin-PEG nanoparticles (Apt-CUR-NPs).^69^ In Apt-CUR-NPs strategy, RNA aptamer specifically targets EpCAM protein in colorectal cancer cell. This nanoparticle is constructed as following: PLGA serves as a hydrophobic core for encapsulating drugs and PEG locates the lipid surface for target ligand modification (Fig. 2). Using nanoprecipitation, Apt-CUR-NPs achieve a particle size of 90.1% ± 4.2%, possessing highly efficient encapsulation. Apt-CUR-NPs increase the ability of curcumin to bind to colon cancer cells, as well as its bioavailability in vivo.^69^

### Aptamer-hyaluronan/chitosan conjugates for targeted drug delivery

MUC1 is overexpressed in colorectal cancer. The targeted-MUC1 aptamer conjugated with hyaluronan/chitosan nanoparticles (HACSNPs) was constructed as a vehicle for the targeted delivery of 5-fluorouracil to colorectal cancer cells (Fig. 2). In this delivery system, chitosan has high solubility in water and is highly mucoadhesive,^70^ HACSNPs show some advantages with high combination and penetration for the drug delivery systems. These nanoparticles are approximately 181 nm with an encapsulation efficiency of 45.5 ± 2.8, and acceptable stability. The aptamer-targeted nanoparticle has a higher specific cytotoxicity to tumor cells, and its IC50 value is significantly decreased (1.43 ± 0.42) with respect to CRCcells.^71^ Thus, this aptamer-hyaluronan/chitosan complex is a promising strategy for efficient delivery to targeted cells and has low toxicity in non-targeted cells.

### Aptamer-mesoporous silica conjugates for targeted drug delivery

Mesoporous silica nanoparticle (MSN) provides superior solid support in delivery systems, which contains mesoporous structure, high rigidity, good biocompatibility, and chemical stability.^72,73^ EpCAM is overexpressed in human colon cancer and initially discovered as an antigen.^60^ The targeted-EpCAM aptamer was selected. This aptamer functional doxorubicin-loaded mesoporous silica nanoparticle (Apt-MSN-DOXs) was effectively used in delivering DOX for the therapy of CRC metastasis (Fig. 2). Additionally, Apt-MSN-DOXs increases the cellular uptake and enhance the cytotoxicity of chemotherapeutic drug DOX to colorectal cancer cells. The drug releasing behavior of Apt-MSN-DOXs shows that they release more drugs in the acidic environment (pH5.5) than in neutral environment (pH7.4).^74^

## Conclusions

Currently, the aptamers identified using cell-SELEX technology have been extensively applied for cancer biomarker discovery, clinical diagnosis, imaging and targeted therapy. Cell-SELEX is an efficient method for selecting aptamers against whole live cells and targeting various cellular surface molecules. However, aptamer selection based on cell-SELEX technique still faces various challenges. Aptamer screening pool and cell-surface molecules are complexity, resulting that aptamer screening is complicated and difficult to standardize in vivo. Moreover, cell-surface molecules especially membrane proteins have not been identified. Cell-SELEX technology requires further to improve aptamer-screening methods in vitro and in vivo, as well as labeling and imaging techniques in vivo. Furthermore, typical SELEX technology is a time-consuming and costly process of aptamer screening. Cell-SELEX strategy is still confronted with automated or high-throughput needs. New technologies based on cell-SELEX, including fluorescence-activated cell sorting (FACS)-SELEX, 3D cell-SELEX, and Hybrid-SELEX, can be used to solve the above problems, such as FACS device is used to separate dead cells,^75^ 3D cell-SELEX is used to imitates tissue microenvironment in vitro,^44^ Hybrid-SELEX strategy generates aptamers against targets in the irnative state on the surface of cells.^76^ In the future, aptamers decorated and conjugated with chemical are served as a significant molecule tool.
